# Improving Quality Assurance in a Radiation Oncology Using ARIA Visual Care Path

**DOI:** 10.3390/jpm14040416

**Published:** 2024-04-14

**Authors:** Ilaria Bonaparte, Federica Fragnoli, Fabiana Gregucci, Roberta Carbonara, Fiorella Cristina Di Guglielmo, Alessia Surgo, Valerio Davì, Morena Caliandro, Giuseppe Sanfrancesco, Christian De Pascali, Alberto Aga, Chiara Indellicati, Rosalinda Parabita, Rosilda Cuscito, Pietro Cardetta, Maurizio Laricchia, Michele Antonicelli, Annarita Ciocia, Domenico Curci, Pietro Guida, Maria Paola Ciliberti, Alba Fiorentino

**Affiliations:** 1 Department of Radiation Oncology, Miulli General Regional Hospital, 70021 Bari, Italy; ilaria.bonaparte@medipass.it (I.B.); federica.fragnoli@pec.tsrm.org (F.F.); f.gregucci@miulli.it (F.G.); roberta.carbonara@miulli.it (R.C.); f.diguglielmo@miulli.it (F.C.D.G.); a.surgo@miulli.it (A.S.); v.davi@miulli.it (V.D.); morenacaliandro@yahoo.it (M.C.); giuseppe.sanfrancesco@pec.tsrm.org (G.S.); alberto.aga@pec.tsrm.org (A.A.); chiara.indellicati@pec.tsrm.org (C.I.); rosalinda.parabita@pec.tsrm.org (R.P.); rosilda.cuscito@pec.tsrm.org (R.C.); pietro-1999@live.it (P.C.); maurizio.laricchia@pec.tsrm.org (M.L.); antonicelli.michele.92@gmail.com (M.A.); annaritaciocia@pec.it (A.C.); domenica.curci7@pec.it (D.C.); p.guida@miulli.it (P.G.); m.ciliberti@miulli.it (M.P.C.); 2 Department of Medicine and Surgery, LUM University, 70010 Bari, Italy

**Keywords:** radiotherapy, quality assurance, care path, workflow

## Abstract

Purpose: Errors and incidents may occur at any point within radiotherapy (RT). The aim of the present retrospective analysis is to evaluate the impact of a customized ARIA Visual Care Path (VCP) on quality assurance (QA) for the RT process. Materials and Methods: The ARIA VCP was implemented in June 2019. The following tasks were customized and independently verified (by independent checks from radiation oncologists, medical physics, and radiation therapists): simulation, treatment planning, treatment start verification, and treatment completion. A retrospective analysis of 105 random and unselected patients was performed, and 945 tasks were reviewed. Patients’ reports were categorized based on treatment years period: 2019–2020 (A); 2021 (B); and 2022–2023 (C). The QA metrics included data for timeliness of task completion and data for minor and major incidents. The major incidents were defined as incorrect prescriptions of RT dose, the use of different immobilization systems during RT compared to the simulation, the absence of surface-guided RT data for patients’ positioning, incorrect dosimetric QA for treatment plans, and failure to complete RT as originally planned. A sample size of approximately 100 was able to obtain an upper limit of 95% confidence interval below 5–10% in the case of zero or one major incident. Results: From June 2019 to December 2023, 5300 patients were treated in our RT department, an average of 1300 patients per year. For the purpose of this analysis, one hundred and five patients were chosen for the study and were subsequently evaluated. All RT staff achieved a 100% compliance rate in the ARIA VCP timely completion. A total of 36 patients were treated in Period A, 34 in Period B, and 35 in Period C. No major incidents were identified, demonstrating a major incident rate of 0.0% (95% CI 0.0–3.5%). A total of 26 out of 945 analyzed tasks (3.8%) were reported as minor incidents: absence of positioning photo in 32 cases, lack of patients’ photo, and absence of plan documents in 4 cases. When comparing periods, incidents were statistically less frequent in Period C. Conclusions: Although the present analysis has some limitations, its outcomes demonstrated that software for the RT workflow, which is fully integrated with both the record-and-verify and treatment planning systems, can effectively manage the patient’s care path. Implementing the ARIA VCP improved the efficiency of the RT care path workflow, reducing the risk of major and minor incidents.

## 1. Introduction

Radiotherapy (RT) is a viable treatment option for patients suffering from cancer, as it can significantly improve their overall survival and quality of life [1]. Modern radiation therapy involves a series of complex procedures, including treatment preparation, planning, and delivery. The process necessitates the involvement of multiple professionals, such as physicians, physicists, and technicians. To ensure the safety of patients and minimize errors, it is crucial to carry out patient care during radiation therapy in an efficient and accurate sequence.

The delivery of high-quality patient care requires the effective sharing of clinical and technical information, timely completion of tasks, and efficient communication among members of the radiotherapy team. Additionally, the seamless integration and proper functioning of advanced equipment and software by the team members is vital for the success of this process [2,3,4]. To ensure the safety and effectiveness of RT, quality checks must be included in the process, using both proactive and reactive measures to minimize the risks of errors and incidents [4,5,6].

As reported in previous studies, errors and incidents may occur at any point within the RT process [7,8]. Over the years, it has been demonstrated that implementing a comprehensive quality assurance (QA) program can detect and correct errors occurring in the complex process of radiotherapy, leading to improved treatment quality for patients [9].

The term ‘incident’ is defined as an event or series of events that could or did lead to a dose error, and not only, in a patient receiving radiation therapy. Studies conducted previously have revealed that the use of an RT workspace and a QA checking program can significantly decrease errors in radiation therapy. These tools help to minimize delays and mistakes, leading to higher levels of patient and staff satisfaction [2,9].

Varian, a company based in Palo Alto, California, has introduced ARIA Visual Care Path (VCP), which is a workflow management tool that is seamlessly integrated with record-and-verify and treatment planning systems. The VCP software enables the standardization and optimization of patient care across all levels.

As recently reported, Kovalchuk et al. [2] conducted an analysis to quantify the impact of ARIA VCP implementation in an RT department on efficiency, safety, and staff satisfaction by comparing three-time phases: pre-VCP, transition, and post-VCP. The authors showed an improvement in workflow efficiency [2].

Our institution has been using ARIA VCP since its inception and regularly upgrades it with annual enhancements. Our quality improvement team, comprising experts from multiple disciplines, has developed customized modules for ARIA VCP that cover all the critical steps involved in patient treatment planning and delivery.

Data in the literature are scarce regarding tool evaluation to improve RT workflow. Thus, we are conducting a retrospective analysis to measure the impact of a customized ARIA VCP implementation on daily clinical practice, specifically in terms of QA in our Department of Radiation Oncology.

## 2. Materials and Methods

### 2.1. VPC Customization

The General Regional Hospital F. Miulli Advanced Radiation Therapy Department (Miulli.art) treats approximately 1200 patients yearly and two Varian linear accelerators (TrueBeam) with more than 25 staff members. The department operates on the ARIA Radiation Oncology platform and the Eclipse treatment planning system (Varian Medical Systems). Clinical activity of Miulli.art started in June 2019, and the Department of Radiation implemented the ARIA VCP to enhance the patient care path RT workflow. A multidisciplinary team consisting of radiation oncologists (ROs), medical physicists (MPs), and radiotherapists (RTTs) worked together to review and customize the process into separate ARIA VCP tasks. These tasks include simulation, treatment planning, treatment start verification, and treatment completion.

**Figure 1:**
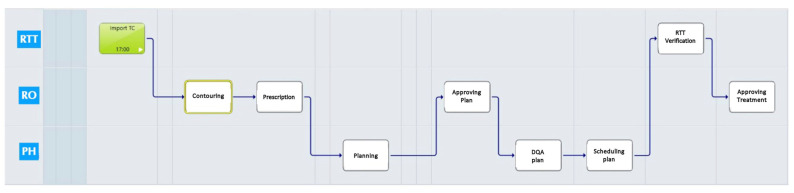
ARIA VCP (Visual Care Path) RT workflow from simulation to treatment completion.

By utilizing ARIA data administration, we were able to efficiently enter the necessary tasks, ultimately leading to the development of a set of ARIA VCP templates as illustrated in Figure 1:

1. Simulation task: It includes a simulation report with patients’ face photos for recognition and a clinical summary; immobilization procedure details and an immobilization system photo; and simulation computed tomography (CT) import.
2. Treatment planning task: Contouring activities, dose prescription, treatment planning, plan approval, and dosimetric quality assurance (DQA) assessment are included.
3. Treatment start verification task: This includes dose prescription and monitor unit review; verification of patients’ RT documents (plan document, simulation document, plan check DQA, DVH, SGRT data procedures, correct immobilization system); and photo review.
4. Treatment completion task: This includes the verification of RT dose and fractions (delivered versus planned).

After each phase, based on the ARIA VPC tasks, a different team member performed an independent check for each patient to minimize the possibility of errors. The control for each independent check was verified with a unique password by different members of the staff. Once approved, the next step in the RT phase can be taken.

After completing the simulation tasks, the dosimetrist (RTT) and MP reviewed and approved them before proceeding to the next phase. After the treatment plan phase, two independent checkers performed a quality control check on contouring, dose prescription, radiation therapy plan, and dosimetric data by RO and MP. At the beginning of therapy, the ROs and RTT performed two independent checks to verify clinical data, plan documents, and simulation documents, and plan to check DQA, DVH, and surface-guided RT (SGRT) data procedures. At the end of treatment, RO verified and checked the RT dose and fractions verification.

### 2.2. Study Procedures

The objective of this retrospective study was to assess the effectiveness of a personalized ARIA VCP implementation in everyday clinical practice concerning quality assurance. A retrospective analysis of 105 randomly selected and unfiltered patients was conducted to achieve this. A total of 945 tasks were reviewed for this purpose. As part of the yearly analysis and solution implementation, ARIA VCP was improved over the course of time; thus, for this reason, patient reports were divided into three treatment year periods: 2019–2020 (Period A), 2021 (Period B), and 2022–2023 (Period C). Quality assurance metrics were used to measure the timeliness of task completion and the occurrence of minor and major incidents. Major incidents were defined as incorrect prescription of RT dose, use of different immobilization systems during RT compared to the simulation, absence of SGRT data for patient positioning, incorrect DQA for treatment plans, and failure to complete RT as originally planned. Minor incidents refer to any other errors not included in the major incidents category.

Approval for the study was not required in accordance with local and national legislation; however, informed consent was obtained from all patients included in the study.

### 2.3. Statistical Analysis

**Figure 2:**
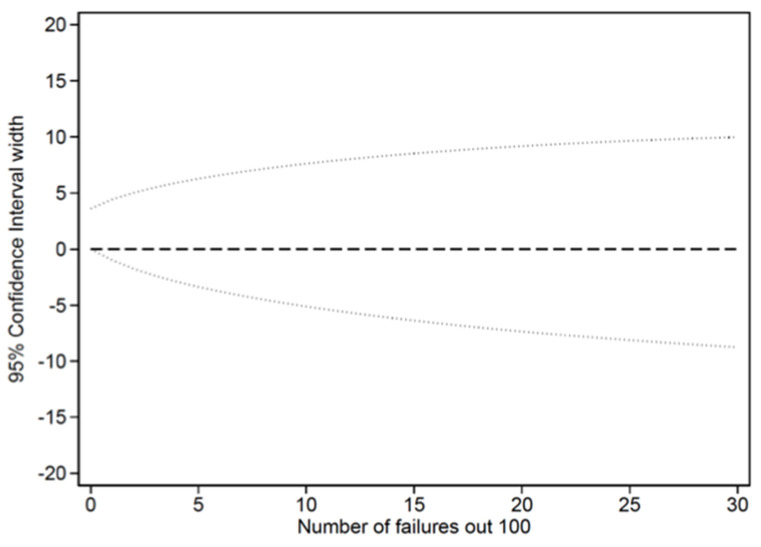
When the sample size is greater than 100, a proportion less than or equal to 1% has a 95% confidence interval less than or equal to 5%, while a proportion less than or equal to 5% has a 95% confidence interval less than or equal to 10%.

Data are reported as mean and standard deviation, or frequency with percentage. Associations between categorical variables were evaluated using the chi-squared or Fisher exact test, as appropriate. Continuous variables were compared using an analysis of variance. A binomial exact 95% confidence interval (95% CI) was estimated. A sample size of approximately one hundred was able to obtain an upper limit of 95% confidence interval below 5–10% in the case of 0 or 1 major incident, as shown in Figure 2. When the sample size exceeds 100, a proportion that is equal to or less than 1% will have a 95% confidence interval of 5% or less, while a proportion that is equal to or less than 5% will have a 95% confidence interval of 10% or less. A Poisson distribution was used to evaluate the total number of incidents, and the incidence rate ratios were calculated to compare periods. A *p*-value of 0.05 or less was considered statistically significant. All analyses were conducted using STATA software, version 16 (Stata-Corp LP, College Station, TX, USA).

## 3. Results

Our department achieved a 100% compliance rate in completing and using simulation preparation ARIA VCP tasks on time for all patients, thanks to the hard work of RO, MP, and RTT.

From June 2019 to December 2023, 5300 patients were treated in our RT department, an average of 1.300 patients per year. For the purpose of this analysis, a random sample of more than 100 patients was selected to assess the risk of incidents. Roughly 35 patients were selected for each period.

One hundred and five patients were chosen for the study and were subsequently evaluated. Patient characteristics are listed in Table 1. The median age of the patients was 65 years, with an age range of 40 to 92. The study group consisted of 47 females and 58 males. Of the patients, 65% received Volumetric Modulated Arc Therapy (VMAT), with an average total dose of 44 Gy delivered in 17 fractions; in comparison, 35% received stereotactic radiotherapy, with an average total dose of 35 Gy delivered in 5 fractions. The most commonly treated cancers were breast, prostate, lung, rectum, brain cancers, and bone metastases, which are all listed in Table 1.

**Figure 3:**
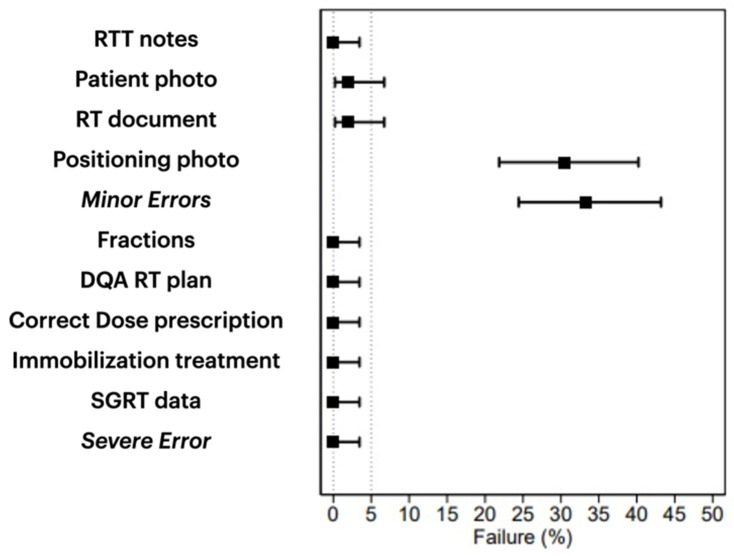
Minor and severe errors plot.

As reported in Table 2, thirty-six patients received treatment in Period A, 34 in Period B, and 35 in Period C. Out of a total of 945 tasks, 38 incidents were reported, which accounts for approximately 4% of all incidents. Among these, two major incidents were identified: RT was not completed (the planned and delivered doses were not the same). Unfortunately, one patient passed away while another experienced grade 4 hematological toxicity, and RT was stopped. However, these incidents were not considered errors. Thus, no major incidents were reported, indicating a major incident rate of 0.0% (95% confidence interval of 0.0–3.5%; Figure 3).

**Figure 4:**
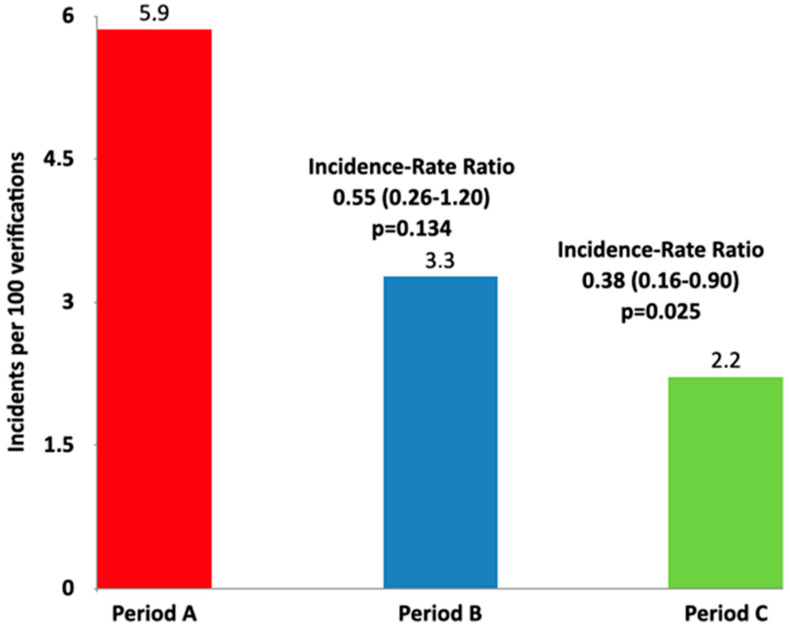
Incidence rate ratio for Periods A, B, and C.

Out of 945 analyzed tasks, 36 incidents were reported as minor. This accounts for 3.8% of incidents, with a confidence interval of 2.8–5.3%. The incidents included the absence of positioning photos in 32 cases, the absence of patients’ photos, and the absence of plan documents in 4 cases. When comparing Period B and C to Period A, incidents were less frequent. The incidence rate ratio for period A/B was 0.55 (95% CI 0.26–1.20) with a *p*-value of 0.134. For period A/C, the incidence rate ratio was 0.38 (95% CI 0.16–0.90) with a *p*-value of 0.025. Please refer to Table 2 and Figure 4 for more information.

Of the 105 cases, 81 patients (77%) began their radiation therapy within ten days of the CT simulation, while the remaining 24 (23%) started their radiation therapy from 11 to 16 working days after the simulation. The radiation oncologist took an average of 5 ± 3 days to complete the treatment contouring and prescriptions, while the medical physicist took an average of 4 ± 2 days to complete the planning.

## 4. Discussion

Over the past few decades, advancements in radiotherapy technologies and techniques have been significant. These advancements have given healthcare professionals the ability to modulate the dose of radiation [10,11]. They have also improved the accuracy of targeting movements through techniques such as deep inspiration breath hold or gating irradiation [12]. Furthermore, radiotherapy is now being used to treat non-oncological diseases, which is an improvement in its utilization [13,14].

According to the International Atomic Energy Agency (IAEA), an incident can be defined as any unexpected event that could lead to equipment malfunctions, operating errors, or other mishaps whose consequences or potential consequences are not negligible from the perspective of safety or protection. The IAEA has gathered and summarized valuable lessons learned from accidental exposures in radiotherapy in order to prevent such incidents from happening in the future [15,16].

It is well established that errors can arise at any stage of the multi-step treatment process, some of which can be identified and prevented prior to treatment, while others may only become apparent during treatment delivery. Although the necessary steps for safely administering radiation therapy to a patient are well-defined, there still needs to be more clarity around the workflow and efficiency mapping of this process [2].

According to Zarei et al. [3], the most common causes of incidents in RT were inadequate direction or information exchange, non-adherence to established standards or procedures, miscommunication, and insufficient planning documentation. Also, Yeung et al., in their analysis of 10 years, showed that the most frequent were documentation errors, which accounted for 42.1% of the incidents [9]. Over 50% of documentation errors resulted from data transfer and communication errors [9].

It is very important for a radiation oncology department to have a well-defined plan in place to prevent incidents and their potential impact on patient safety. To avoid errors, it is essential to identify the common causes and nature of near-miss situations and evaluate the effectiveness of independent checks [17,18]. In the management of patient care safety in radiation therapy, it is widely recognized that analyzing incidents and potential errors can enhance patient safety and reduce the likelihood of recurring issues [17,18,19,20].

Our department utilized ARIA VCP vers. 11–18, a workflow tool fully integrated with both the record-and-verify and treatment planning systems, to manage the patient’s care path. In order to evaluate the quality assurance of RT workflow, a retrospective analysis was conducted, reviewing the records of 105 patients to demonstrate the absence of severe incidents.

All 105 patients received modern radiotherapy (VMAT and stereotactic RT), which is considered a complex treatment for those who adhere to established protocols and guidelines, which is crucial in reducing the likelihood of incidents. Out of 105 patients and 945 evaluated tasks, no severe incidents were documented, showing that the workflow ARIA VCP, customized for our center, is helpful for a quality assurance process in RT patients’ care paths.

Regarding minor incidents, we reported 36 out of 945 errors with a rate of 3.8 (2.8–5.3) per 100 verifications. In 89% of cases (32 patients/105), the minor incident consisted of the absence of a positioning photo. Analyzing tasks divided by age, we noted that incidents were less frequent in periods B and C: there were 18 minor incidents during period A, 10 in period B, and 7 in period C (*p* 0.023). This reduction in minor incidents was obtained by customizing VCP and safety barriers and by regular independent chart checking. In fact, in 2021, the independent check by RTT (before treatment started) was introduced. This last safety barrier is essential due to other experiences that enhanced the fact that almost 65.6% of the incidents in the final study population were discovered by RTT [3]. A group of researchers analyzed radiotherapy incidents, reporting that the majority of incidents were declared by RTT (86%). In comparison, only 2.4% and 11.6% of the incidents were reported by oncologists and medical physicists, respectively [21].

The publication of the Radiation Oncology Safety Information System [22] highlighted the significance of staff vigilance, particularly the RTT, in detecting incidents at the treatment unit. As they are directly involved in delivering treatment and are near the patient, they play a critical role in identifying potential safety hazards. Therefore, all the analyses mentioned above stress the importance of RT staff’s participation in promoting a “safety culture.”

Moreover, previous investigations have shown that errors in radiation therapy can be reduced by the implementation of a quality assurance checking program [9]. According to a study conducted by Yeung et al., a significant number of incidents (about 59%) related to errors in documentation and treatment planning were detected by performing checks prior to the commencement of the treatment course.

The data presented here are comparable to the results reported by Yeung et al. [9]. In fact, their study has shown that the checks performed by physicists and dosimetrists on the treatment plan and the first patient setup were highly influential in detecting errors in documentation and treatment planning. This emphasizes the importance of our quality program. In our department with ARIA VCP, we conduct a customized independent check at the end of every task and procedure to ensure that each step is controlled effectively.

Regarding ARIA VCP data, only one experience/analysis was published in 2015.

The authors provided interactive flow charts outlining both serial and parallel tasks assigned to specific parties and scheduled at set intervals, which are similar to the ones reported in the present analysis [2]. They showed a comparison between the pre- and post-ARIA VCP eras and noted that there was a two-fold increase in the rate of timely completion of tasks. In our analysis, we found that the contouring/prescription task took five days to complete, while the planning preparation phase took four days. According to Kovalchuk et al., before the implementation of ARIA VCP, physicians’ treatment prescriptions were completed an average of 1.4 ± 9.3 days late. However, after the implementation of ARIA VCP, they were completed on average 5.6 ± 6.3 days early. The introduction of ARIA VCP also reduced the number of errors detected during the physics plan check. The authors reported a monthly defect rate of 5.2% ± 4.1% with ARIA VCP, compared to the rate of 19.1% ± 1.3% without ARIA VCP. Notably, there were no errors that propagated to block verification.

In terms of errors, no severe or significant incidents were documented in the present analysis. The minor error rate was 3.8 (2.8–5.3) per 100 verifications, aligning with Kovalchuk et al.’s findings. The rate decreased over the three analyzed periods due to the department’s implementation of a quality assurance protocol based on the Define/Measure/Analyze/Improve/Control (DMAIC) approach. This approach is a data-driven improvement cycle used for optimizing, improving, and stabilizing industry processes [23]. Every year, trends are analyzed, and solutions are implemented in the newly developed ARIA VCP.

### Limitations of the Study

It is important to acknowledge that the present analysis has some limitations. These include the retrospective nature of the study, the definition of error as either minor or severe, the heterogeneity of the sample size in terms of tumors, radiation therapy sites, doses, and techniques, as well as the small sample size analyzed.

## 5. Conclusions

To summarize, an incident report system during RT workflow has proven to be an effective tool for documenting and categorizing incidents, evaluating the impact of incidents on patients regarding dosage errors, and assessing the efficacy of QA checks. It is important to note that human errors are a possibility during various stages of radiation therapy. If left uncorrected, these errors could lead to significant dosage errors that could harm patients. Implementing a quality assurance checking program can significantly reduce errors, though it can never completely eliminate them. Ensuring that staff receive ongoing education is a crucial factor in detecting errors early on in radiation therapy. This plays an essential role in providing quality patient care. Although the present analysis has some limitations, its outcomes demonstrated that software for the RT workflow, which is fully integrated with both the record-and-verify and treatment planning systems, can effectively manage the patient’s care path. The ARIA VCP, with multiple safety barriers, can be very helpful in preventing incidents, improving task completion, reducing near misses, and enhancing communication. The ARIA VCP minimizes the risk of errors and promotes a more organized and safer work setting. Advanced radiation technologists should enhance safety measures to reduce the possibility of incidents and improve the QA in RT [24].

## Figures and Tables

**Table 1:** Patients characteristic.

		Period A	Period B	Period C
Number of Patients				
		36	34	35
Gender				
	Male	24 (66.7%)	14 (41.2%)	20 (57.1%)
	Female	12 (33.3%)	20 (58.8%)	15 (42.9%)
Age				
	Median (IQR) (years)	67 (28–82)	63 (40–83)	64 (38–92)
RT Sites				
	Brain	6 (16.7%)	4 (11.8%)	2 (5.7%)
	Bone metastases	7 (19.4%)	3 (8.8%)	8 (22.9%)
	Breats	6 (16.7%)	9 (26.5%)	7 (20%)
	Prostate	6 (16.7%)	2 (5.9%)	6 (17.1%)
	Lung	3 (8.3%)	3 (8.8%)	3 (8.6%)
	Pelvis (rectum/gynecological cancer)	1 (2.8%)	1 (2.9%)	2 (5.7%)
	Others	7 (19.4%)	12 (35.3%)	7 (20%)
RT Doses/Techniques				
	SBRT (1–3 fx)	6 (16.7%)	1 (2.9%)	4 (11.4%)
	SBRT (5–8 fr)	8 (22.2%)	9 (26.5%)	8 (22.8%)
	RT ≤ 15 fr	11 (30.55%)	12 (35.3%)	13 (37.2%)
	RT > 15 fr	11 (30.55%)	12 (35.3%)	10 (28.6%)

RT: radiotherapy; SBRT: stereotactic radiotherapy; fr: fractions.

**Table 2:** Minor and major incidents for periods (A, B, and C).

	All Patients	Period A	Period B	Period C	
	*n* = 105	*n* = 36	*n* = 34	*n* = 35	*p*
Dose Prescription	0 (0.0%)	0 (0.0%)	0 (0.0%)	0 (0.0%)	
Patients photo	2 (1.9%)	1 (2.8%)	0 (0.0%)	1 (2.9%)	1.000
Treatments note	0 (0.0%)	0 (0.0%)	0 (0.0%)	0 (0.0%)	
Positioning photo	32 (30.5%)	17 (47.2%)	10 (29.4%)	5 (14.3%)	0.010
Immobilization system	0 (0.0%)	0 (0.0%)	0 (0.0%)	0 (0.0%)	
SGRT data	0 (0.0%)	0 (0.0%)	0 (0.0%)	0 (0.0%)	
Treatment documents	2 (1.9%)	1 (2.8%)	0 (0.0%)	1 (2.9%)	1.000
DQA RT plan	0 (0.0%)	0 (0.0%)	0 (0.0%)	0 (0.0%)	
Number of fractions	0 (0.0%)	0 (0.0%)	0 (0.0%)	0 (0.0%)	
Minor incidents	35 (33.3%)	18 (50.0%)	10 (29.4%)	7 (20.0%)	0.023
Severe incidents	0 (0.0%)	0 (0.0%)	0 (0.0%)	0 (0.0%)	

## Data Availability

Data are available to corresponding author.

## Abbreviations

RT	Radiotherapy
QA	Quality Assurance
VCP	Visual Care Path
RO	Radiation Oncologists
MP	Medical Physicists
RTT	Radiotherapists
DQA	Dosimetric Quality Assurance
SGRT	Surface-Guided RT
VMAT	Volumetric Modulated Arc Therapy
IAEA	International Atomic Energy Agency
